# The advantages of the Matthews correlation coefficient (MCC) over F1 score and accuracy in binary classification evaluation

**DOI:** 10.1186/s12864-019-6413-7

**Published:** 2020-01-02

**Authors:** Davide Chicco, Giuseppe Jurman

**Affiliations:** 1 Krembil Research Institute, Toronto, Ontario, Canada; 2 Peter Munk Cardiac Centre, Toronto, Ontario, Canada; 3 Fondazione Bruno Kessler, Trento, Italy

**Keywords:** Matthews correlation coefficient, Binary classification, F_1_ score, Confusion matrices, Machine learning, Biostatistics, Accuracy, Dataset imbalance, Genomics

## Abstract

**Background:**

To evaluate binary classifications and their confusion matrices, scientific researchers can employ several statistical rates, accordingly to the goal of the experiment they are investigating. Despite being a crucial issue in machine learning, no widespread consensus has been reached on a unified elective chosen measure yet. Accuracy and F_1_ score computed on confusion matrices have been (and still are) among the most popular adopted metrics in binary classification tasks. However, these statistical measures can dangerously show overoptimistic inflated results, especially on imbalanced datasets.

**Results:**

The Matthews correlation coefficient (MCC), instead, is a more reliable statistical rate which produces a high score only if the prediction obtained good results in all of the four confusion matrix categories (true positives, false negatives, true negatives, and false positives), proportionally both to the size of positive elements and the size of negative elements in the dataset.

**Conclusions:**

In this article, we show how MCC produces a more informative and truthful score in evaluating binary classifications than accuracy and F_1_ score, by first explaining the mathematical properties, and then the asset of MCC in six synthetic use cases and in a real genomics scenario. We believe that the Matthews correlation coefficient should be preferred to accuracy and F_1_ score in evaluating binary classification tasks by all scientific communities.

## Background

Given a clinical feature dataset of patients with cancer traits [1, 2], which patients will develop the tumor, and which will not? Considering the gene expression of neuroblastoma patients [3], can we identify which patients are going to survive, and which will not? Evaluating the metagenomic profiles of patients [4], is it possible to discriminate different phenotypes of a complex disease? Answering these questions is the aim of machine learning and computational statistics, nowadays pervasive in analysis of biological and health care datasets, and many other scientific fields. In particular, these binary classification tasks can be efficiently addressed by supervised machine learning techniques, such as artificial neural networks [5], *k*-nearest neighbors [6], support vector machines [7], random forest [8], gradient boosting [9], or other methods. Here the word *binary* means that the data element statuses and prediction outcomes (class labels) can be twofold: in the example of patients, it can mean healthy/sick, or low/high grade tumor. Usually scientists indicate the two classes as the negative and the positive class. The term *classification* means that the goal of the process is to attribute the correct label to each data instance (sample); the process itself is known as the classifier, or classification algorithm.

Scientists have used binary classification to address several questions in genomics in the past, too. Typical cases include the application of machine learning methods to microarray gene expressions [10] or to single-nucleotide polymorphisms (SNPs) [11] to classify particular conditions of patients. Binary classification can also be used to infer knowledge about biology: for example, computational intelligence applications to ChIP-seq can predict transcription factors [12], applications to epigenomics data can predict enhancer-promoter interactions [13], and applications to microRNA can predict genomic inverted repeats (pseudo-hairpins) [14].

A crucial issue naturally arises, concerning the outcome of a classification process: how to evaluate the classifier performance? A relevant corpus of published works has stemmed until today throughout the last decades for possible alternative answers to this inquiry, by either proposing a novel measure or comparing a subset of existing ones on a suite of benchmark tasks to highlight pros and cons [15–28], also providing off-the-shelf software packages [29, 30]. Despite the amount of literature dealing with this problem, this question is still an open issue. However, there are several consolidated and well known facts driving the choice of evaluating measures in the current practice.

**Accuracy, MCC, F**_**1**_** score**. Many researchers think the most reasonable performance metric is the ratio between the number of correctly classified samples and the overall number of samples (for example, [31]). This measure is called *accuracy* and, by definition, it also works when labels are more than two (multiclass case). However, when the dataset is unbalanced (the number of samples in one class is much larger than the number of samples in the other classes), accuracy cannot be considered a reliable measure anymore, because it provides an overoptimistic estimation of the classifier ability on the majority class [32–35].

An effective solution overcoming the class imbalance issue comes from the *Matthews correlation coefficient (MCC)*, a special case of the *ϕ* phi coefficient [36].

Stemming from the definition of the phi coefficient, a number of metrics have been defined and mainly used for purposes other than classification, for instance as association measures between (discrete) variables, with the *Cramér’s V* (or *Cramér’s* *ϕ*) being one of the most common rates [37].

Originally developed by Matthews in 1975 for comparison of chemical structures [38], MCC was re-proposed by Baldi and colleagues [39] in 2000 as a standard performance metric for machine learning with a natural extension to the multiclass case [40]. MCC soon started imposing as a successful indicator: for instance, the Food and Drug Administration (FDA) agency of the USA employed the MCC as the main evaluation measure in the MicroArray II / Sequencing Quality Control (MAQC/SEQC) projects [41, 42]. The effectiveness of MCC has been shown in other scientific fields as well [43, 44].

Although being widely acknowledged as a reliable metric, there are situations - albeit extreme - where either MCC cannot be defined or it displays large fluctuations [45], due to imbalanced outcomes in the classification. Even if mathematical workarounds and Bayes-based improvements [46] are available for these cases, they have not been adopted widely yet.

Shifting context from machine learning to information retrieval, and thus interpreting positive and negative class as relevant and irrelevant samples respectively, the recall (that is the accuracy on the positive class) can be seen as the fraction of relevant samples that are correctly retrieved. Then its dual metric, the precision, can be defined as the fraction of retrieved documents that are relevant. In the learning setup, the pair precision/recall provides useful insights on the classifier’s behaviour [47], and can be more informative than the pair specificity/sensitivity [48]. Meaningfully combining precision and recall generates alternative performance evaluation measures. In particular, their harmonic mean has been originally introduced in statistical ecology by Dice [49] and Sørensen [50] independently in 1948, then rediscovered in the 1970s in information theory by van Rijsbergen [51, 52] and finally adopting the current notation of *F*_1_ measure in 1992 [53]. In the 1990s, in fact, *F*_1_ gained popularity in the machine learning community, to the point that it was also re-introduced later in the literature as a novel measure [54].

Nowadays, the *F*_1_ measure is widely used in most application areas of machine learning, not only in the binary scenario, but also in multiclass cases. In multiclass cases, researchers can employ the *F*_1_ micro/macro averaging procedure [55–60], which can be even targeted for *ad-hoc* optimization [61].

The distinctive features of *F*_1_ score have been discussed in the literature [62–64]. Two main properties characterize *F*_1_ from MCC. First, *F*_1_ varies for class swapping, while MCC is invariant if the positive class is renamed negative and vice versa. This issue can be overcome by extending the macro/micro averaging procedure to the binary case itself [17], by defining the *F*_1_ score both on the positive and negative classes and then average the two values (macro), and using the average sensitivity and average precision values (micro). The micro/macro averaged *F*_1_ is invariant for class swapping and its behaviour is more similar to MCC. However, this procedure is biased [65], and it is still far from being accepted as a standard practice by the community. Second, *F*_1_ score is independent from the number of samples correctly classified as negative. Recently, several scientists highlighted drawbacks of the *F*_1_ measure [66, 67]: in fact, Hand and Peter [68] claim that alternative measures should be used instead, due to its major conceptual flaws. Despite the criticism, *F*_1_ remains one of the most widespread metrics among researchers. For example, when Whalen and colleagues released *TargetFinder*, a tool to predict enhancer-promoters interactions in genomics, they showed its results measured only by *F*_1_ score [13], making it impossible to detect the actual true positive rate and true negative rate of their tests [69].

**Alternative metrics**. The current most popular and widespread metrics include *Cohen’s kappa* [70–72]: originally developed to test inter-rater reliability, in the last decades Cohen’s kappa entered the machine learning community for comparing classifiers’ performances. Despite its popularity, in the learning context there are a number of issues causing the kappa measure to produce unreliable results (for instance, its high sensitivity to the distribution of the marginal totals [73–75]), stimulating research for more reliable alternatives [76]. Due to these issues, we chose not to include Cohen’s kappa in the present comparison study.

In the 2010s, several alternative novel measures have been proposed, either to tackle a particular issue such as imbalance [34, 77], or with a broader purpose. Among them, we mention the *confusion entropy* [78, 79], a statistical score comparable with MCC [80], and the *K measure* [81], a theoretically grounded measure that relies on a strong axiomatic base.

In the same period, Powers proposed *informedness* and *markedness* to evaluate binary classification confusion matrices [22]. Powers defines informedness as *true positive rate – true negative rate*, to express how the predictor is informed in relation to the opposite condition [22]. And Powers defines markedness as *precision – negative predictive value*, meaning the probability that the predictor correctly marks a specific condition [22].

Other previously introduced rates for confusion matrix evaluations are *macro average arithmetic* (MAvA) [18], *geometric mean* (Gmean or G-mean) [82], and *balanced accuracy* [83], which all represent classwise weighted accuracy rates.

Notwithstanding their effectiveness, all the aforementioned measures have not yet achieved such a diffusion level in the literature to be considered solid alternatives to MCC and *F*_1_ score. Regarding MCC and *F*_1_, in fact, Dubey and Tatar [84] state that these two measure “provide more realistic estimates of real-world model performance”.

However, there are many instances where MCC and *F*_1_ score disagree, making it difficult for researchers to draw correct deductions on the behaviour of the investigated classifier.

MCC, *F*_1_ score, and accuracy can be computed when a specific statistical threshold *τ* for the confusion matrix is set. When the confusion matrix threshold is not unique, researchers can instead take advantage of classwise rates: *true positive rate* (or *sensitivity*, or *recall*) and *true negative rate* (or *specificity*), for example, computed for all the possible confusion matrix thresholds. Different combinations of these two metrics give rise to alternative measures: among them, the area under the *receiver operating characteristic curve* (AUROC or ROC AUC) [85–91] plays a major role, being a popular performance measure when a singular threshold for the confusion matrix is unavailable. However, ROC AUC presents several flaws [92], and it is sensitive to class imbalance [93]. Hand and colleagues proposed improvements to address these issues [94], that were partially rebutted by Ferri and colleagues [95] some years later.

Similar to ROC curve, the *precision-recall (PR) curve* can be used to test all the possible positive predictive values and sensitivities obtained through a binary classification [96]. Even if less common than the ROC curve, several scientists consider the PR curve more informative than the ROC curve, especially on imbalanced biological and medical datasets [48, 97, 98].

If no confusion matrix threshold is applicable, we suggest the readers to evaluate their binary evaluations by checking both the PR AUC and the ROC AUC, focusing on the former [48, 97]. If a confusion matrix threshold is at disposal, instead, we recommend the usage of the Matthews correlation coefficient over *F*_1_ score, and accuracy.

In this manuscript, we outline the advantages of the Matthews correlation coefficient by first describing its mathematical foundations and its competitors accuracy and F_1_ score (“Notation and mathematical foundations” section), and by exploring their relationships afterwards (Relationships between rates). We decided to focus on accuracy and F_1_ score because they are the most common metrics used for binary classification in machine learning. We then show some examples to illustrate why the MCC is more robust and reliable than *F*_1_ score, on six synthetic scenarios (“Use cases” section) and a real genomics application (“Genomics scenario: colon cancer gene expression” section). Finally, we conclude the manuscript with some take-home messages (“Conclusions” section).

## Methods

### Notation and mathematical foundations

**Setup**. The framework where we set our investigation is a machine learning task requiring the solution of binary classification problem. The dataset describing the task is composed by *n*^+^ examples in one class, labeled *positive*, and *n*^−^ examples in the other class, called *negative*. For instance, in a biomedical case control study, the healthy individuals are usually labelled negative, while the positive label is usually attributed to the sick patients. As a general practice, given two phenotypes, the positive class corresponds to the *abnormal* phenotype. This ranking is meaningful for example, in different stages of a tumor.

The classification model forecasts the class of each data instance, attributing to each sample its predicted label (positive or negative): thus, at the end of the classification procedure, every sample falls in one of the following four cases:

- Actual positives that are correctly predicted positives are called *true positives* (TP);
- Actual positives that are wrongly predicted negatives are called *false negatives* (FN);
- Actual negatives that are correctly predicted negatives are called *true negatives* (TN);
- Actual negatives that are wrongly predicted positives are called *false positives* (FP).

This partition can be presented in a 2×2 table called *confusion matrix*$\textbf {M}=\left (\begin {array}{ll} \text {TP} & \text {FN}\\ \text {FP} & \text {TN}\end {array}\right)$ (expanded in Table 1), which completely describes the outcome of the classification task.

**Table 1:** The standard confusion matrix M

	Predicted positive	Predicted negative
Actual positive	True positives TP	False negatives FN
Actual negative	False positives FP	True negatives TN

True positives (TP) and true negatives (TN) are the correct predictions, while false negatives (FN) and false positives (FP) are the incorrect predictions

Clearly TP+FN=*n*^+^ and TN+FP=*n*^−^. When one performs a machine learning binary classification, she/he hopes to see a high number of true positives (TP) and true negatives (TN), and less false negatives (FN) and false positives (FP). When $\textbf {M}=\left (\begin {array}{cc}n^{+} & 0\\ 0 & n^{-}\end {array}\right)$the classification is perfect.

Since analyzing all the four categories of the confusion matrix separately would be time-consuming, statisticians introduced some useful statistical rates able to immediately describe the quality of a prediction [22], aimed at conveying into a single figure the structure of **M**. A set of these functions act classwise (either actual or predicted), that is, they involve only the two entries of **M** belonging to the same row or column (Table 2). We cannot consider such measures fully informative because they use only two categories of the confusion matrix [39].

**Table 2:** Classwise performance measures

Sensitivity, recall, true positive rate	${= \frac {\text {TP}}{\text {TP}+\text {FN}} = \frac {\text {TP}}{n^{+}}}$	Specificity, true negative rate	${= \frac {\text {TN}}{\text {TN}+\text {FP}}= \frac {\text {TN}}{n^{-}}}$
Positive predictive value, precision	${= \frac {\text {TP}}{\text {TP}+\text {FP}}}$	Negative predictive value	${= \frac {\text {TN}}{\text {TN}+\text {FN}}}$
False positive rate, fallout	${= \frac {\text {FP}}{\text {FP}+\text {TN}} = \frac {\text {FP}}{n^{-}}}$	False discovery rate	${= \frac {\text {FP}}{\text {FP}+\text {TP}}}$

TP: true positives. TN: true negatives. FP: false positives. FN: false negatives

**Accuracy**. Moving to global metrics having three or more entries of **M** as input, many researchers consider computing the accuracy as the standard way to go. Accuracy, in fact, represents the ratio between the correctly predicted instances and all the instances in the dataset:

1 $$ \text{accuracy} = \frac{\text{TP}+\text{TN}}{n^{+} + n^{-}} = \frac{\text{TP}+\text{TN}}{\text{TP}+\text{TN}+\text{FP}+\text{FN}}   $$

(worst value: 0; best value: 1)

By definition, the accuracy is defined for every confusion matrix **M** and ranges in the real unit interval [0,1]; the best value 1.00 corresponds to perfect classification $\textbf {M}=\left (\begin {array}{cc}n^{+} & 0 \\ 0 & n^{-} \end {array}\right)$ and the worst value 0.00 corresponds to perfect misclassification $\textbf {M}=\left (\begin {array}{cc}0 & n^{+} \\ n^{-} & 0\end {array}\right)$.

As anticipated (Background), accuracy fails in providing a fair estimate of the classifier performance in the class-unbalanced datasets. For any dataset, the proportion of samples belonging to the largest class is called the *no-information error rate*$\text {ni}=\frac {\max \{n^{+},n^{-}\}}{n^{+} + n^{-}}$; a binary dataset is (perfectly) balanced if the two classes have the same size, that is, $\text {ni}=\frac {1}{2}$, and it is unbalanced if one class is much larger than the other, that is $\text {ni}\gg \frac {1}{2}$. Suppose now that $\text {ni}\not = \frac {1}{2}$, and apply the trivial majority classifier: this algorithm learns only which is the largest class in the training set, and attributes this label to all instances. If the largest class is the positive class, the resulting confusion matrix is $\textbf {M}=\left (\begin {array}{cc}n^{+} & 0 \\ n^{-} & 0 \end {array}\right)$, and thus accuracy=ni. If the dataset is highly unbalanced, ni≈1, and thus the accuracy measure gives an unreliable estimation of the goodness of the classifier. Note that, although we achieved this result by mean of the trivial classifier, this is quite a common effect: as stated by Blagus and Lusa [99], several classifiers are biased towards the largest class in unbalanced studies.

Finally, consider another trivial algorithm, the coin tossing classifier: this classifier randomly attributes to each sample, the label positive or negative with probability $\frac {1}{2}$. Applying the coin tossing classifier to any binary dataset gives an accuracy with expected value $\frac {1}{2}$, since $\langle \textbf {M}\rangle = \left (\begin {array}{cc}n^{+} / 2 & n^{+} / 2 \\ n^{-} / 2 & n^{-} / 2\end {array}\right)$.

**Matthews correlation coefficient (MCC)**. As an alternative measure unaffected by the unbalanced datasets issue, the Matthews correlation coefficient is a contingency matrix method of calculating the *Pearson product-moment correlation coefficient* [22] between actual and predicted values. In terms of the entries of **M**, MCC reads as follows:

2 $$ {\begin{aligned} \textrm{MCC} = \frac{\text{TP}\cdot\text{TN}-\text{FP}\cdot\text{FN}}{\sqrt{ (\text{TP}+\text{FP})\cdot(\text{TP}+\text{FN})\cdot(\text{TN}+\text{FP})\cdot(\text{TN}+\text{FN}) }}\  \end{aligned}}  $$

(worst value: –1; best value: +1)

MCC is the only binary classification rate that generates a high score only if the binary predictor was able to correctly predict the majority of positive data instances and the majority of negative data instances [80, 97].

It ranges in the interval [−1,+1], with extreme values –1 and +1 reached in case of perfect misclassification and perfect classification, respectively, while MCC=0 is the expected value for the coin tossing classifier.

A potential problem with MCC lies in the fact that MCC is undefined when a whole row or column of **M** is zero, as it happens in the previously cited case of the trivial majority classifier. However, some mathematical considerations can help meaningfully fill in the gaps for these cases. If **M** has only one non-zero entry, this means that all samples in the dataset belong to one class, and they are either all correctly (for TP≠0 or TN≠0) or incorrectly (for FP≠0 or FN≠0) classified. In this situations, MCC=1 for the former case and MCC=−1 for the latter case. We are then left with the four cases where a row or a column of **M** are zero, while the other two entries are non zero. That is, when **M** is one of $\left (\begin {array}{ll}a & 0 \\ b & 0 \end {array}\right)$, $\left (\begin {array}{ll}a & b \\ 0 & 0 \end {array}\right)$, $\left (\begin {array}{ll}0 & 0 \\ b & a \end {array}\right)$or $\left (\begin {array}{ll}0 & b \\ 0 & a \end {array}\right)$, with *a*,*b*≥1: n in all four cases, MCC takes the indefinite form $\frac {0}{0}$. To detect a meaningful value of MCC for these four cases, we proceed through a simple approximation via a calculus technique. If we substitute the zero entries in the above matrices with the arbitrarily small value *ε*, in all four cases, we obtain

3 $$ \begin{aligned} \textrm{MCC} &= \frac{a\epsilon-b\epsilon}{\sqrt{(a+b)(a+\epsilon)(b+\epsilon)(\epsilon+\epsilon)}}\\ &= \frac{\epsilon}{\sqrt{\epsilon}} \frac{a-b}{\sqrt{2(a+b)(a+\epsilon)(b+\epsilon)}}\\ &\approx \sqrt{\epsilon} \frac{a-b}{\sqrt{2ab(a-b)}} \to 0 \quad \textrm{for \(\epsilon\to 0\)}\ \end{aligned}  $$

With these positions MCC is now defined for all confusion matrices **M**. As a consequences, MCC=0 for the trivial majority classifier, and 0 is also the expected value for the coin tossing classifier.

Finally, in some cases it might be useful to consider the *normalized* MCC, defined as $\textrm {nMCC}=\frac {\textrm {MCC}+1}{2}$, and linearly projecting the original range into the interval [0,1], with $\textrm {nMCC}=\frac {1}{2}$ as the average value for the coin tossing classifier.

**F**_**1**_** score**. This metric is the most used member of the parametric family of the *F*-measures, named after the parameter value *β*=1. *F*_1_ score is defined as the harmonic mean of precision and recall (Table 2) and as a function of **M**, has the following shape:

4 $$ F_{1} \; \text{score} = \frac{2 \cdot \text{TP}}{2 \cdot \text{TP} + \text{FP} + \text{FN}} = 2 \cdot \frac{\text{precision} \cdot \text{recall}}{\text{precision} + \text{recall}}\   $$

(worst value: 0; best value: 1)

*F*_1_ ranges in [0,1], where the minimum is reached for TP=0, that is, when all the positive samples are misclassified, and the maximum for FN=FP=0, that is for perfect classification. Two main features differentiate *F*_1_ from MCC and accuracy: *F*_1_ is independent from TN, and it is not symmetric for class swapping.

*F*_1_ is not defined for confusion matrices $\textbf {M} = \left (\begin {array}{cc}0 & 0 \\ 0 & n^{-} \end {array}\right)$: we can set *F*_1_=1 for these cases. It is also worth mentioning that, when defining the *F*_1_ score as the harmonic mean of precision and recall, the cases TP=0, FP>0, and FN>0 remain undefined, but using the expression $\frac {2 \cdot \text {TP}}{2 \cdot \text {TP} + \text {FP} + \text {FN}}$, the *F*_1_ score is defined even for these confusion matrices and its value is zero.

When a trivial majority classifier is used, due to the asymmetry of the measure, there are two different cases: if *n*^+^>*n*^−^, then $\textbf {M}=\left (\begin {array}{cc}n^{+} & 0 \\ n^{-} & 0 \end {array}\right)$and $F_{1}=\frac {2n^{+}}{2n^{+}n^{-}}$, while if *n*^−^>*n*^+^ then $\textbf {M}=\left (\begin {array}{cc}0 & n^{+} \\ 0& n^{-} \end {array}\right)$, so that *F*_1_=0. Further, for the coin tossing algorithm, the expected value is $F_{1} = \frac {2n^{+}}{3n^{+} + n^{-}}$.

### Relationship between measures

After having introduced the statistical background of Matthews correlation coefficient and the other two measures to which we compare it (accuracy and *F*_1_ score), we explore here the correlation between these three rates. To explore these statistical correlations, we take advantage of the Pearson correlation coefficient (PCC) [100], which is a rate particularly suitable to evaluate the linear relationship between two continuous variables [101]. We avoid the usage of rank correlation coefficients (such as Spearman’s *ρ* and Kendall’s *τ* [102]) because we are not focusing on the ranks for the two lists.

For a given positive integer *N*≥10, we consider all the possible $\binom {N+3}{3}$ confusion matrices for a dataset with *N* samples and, for each matrix, compute the accuracy, MCC and *F*_1_ score and then the Pearson correlation coefficient for the three set of values. MCC and accuracy resulted strongly correlated, while the Pearson coefficient is less than 0.8 for the correlation of *F*_1_ with the other two measures (Table 3). Interestingly, the correlation grows with *N*, but the increments are limited.

**Table 3:** Correlation between MCC, accuracy, and *F*_1_ score values

N	PCC (MCC, *F*_1_ score)	PCC (MCC, accuracy)	PCC (accuracy, *F*_1_ score)
10	0.742162	0.869778	0.744323
25	0.757044	0.893572	0.760708
50	0.766501	0.907654	0.769752
75	0.769883	0.912530	0.772917
100	0.771571	0.914926	0.774495
200	0.774060	0.918401	0.776830
300	0.774870	0.919515	0.777595
400	0.775270	0.920063	0.777976
500	0.775509	0.920388	0.778201
1 000	0.775982	0.921030	0.778652

Pearson correlation coefficient (PCC) between accuracy, MCC and *F*_1_ score computed on all confusion matrices with given number of samples N

**Fig. 1:**
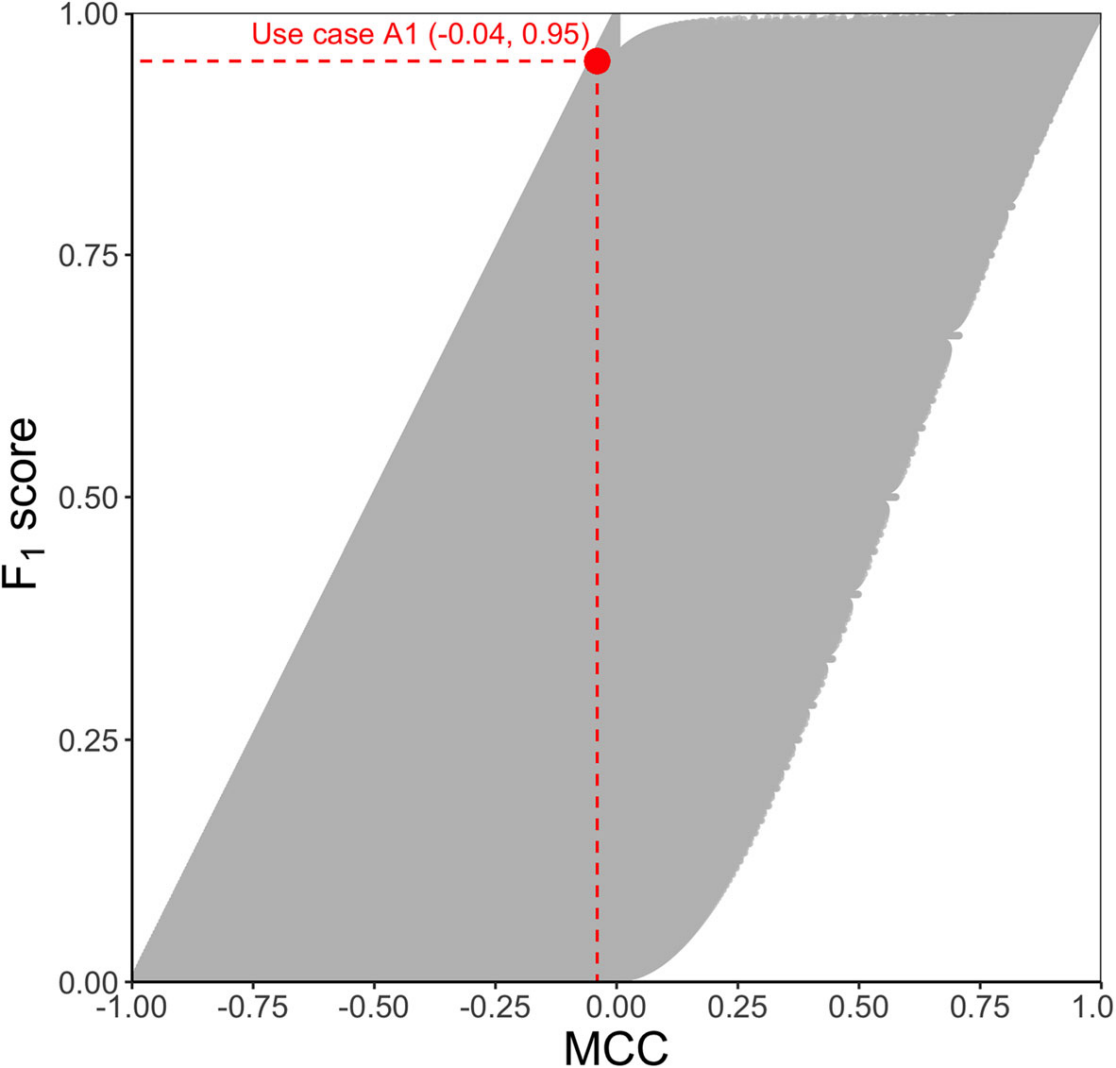
Relationship between MCC and *F*_1_ score. Scatterplot of all the 21 084 251 possible confusion matrices for a dataset with 500 samples on the MCC/ *F*_1_ plane. In red, the (−0.04, 0.95) point corresponding to use case A1

Similar to what Flach and colleagues did for their isometrics strategy [66], we depict a scatterplot of the MCCs and *F*_1_ scores for all the 21 084 251 possible confusion matrices for a toy dataset with 500 samples (Fig. 1). We take advantage of this scatterplot to overview the mutual relations between MCC and *F*_1_ score.

The two measures are reasonably concordant, but the scatterplot cloud is wide, implying that for each value of *F*_1_ score there is a corresponding range of values of MCC and vice versa, although with different width. In fact, for any value *F*_1_=*ϕ*, the MCC varies approximately between [*ϕ*−1,*ϕ*], so that the width of the variability range is 1, independent from the value of *ϕ*. On the other hand, for a given value MCC=*μ*, the *F*_1_ score can range in [0,*μ*+1] if *μ*≤0 and in [*μ*,1] if *μ*>0, so that the width of the range is 1−|*μ*|, that is, it depends on the MCC value *μ*.

Note that a large portion of the above variability is due to the fact that *F*_1_ is independent from TN: in general, all matrices $\textbf {M}=\left (\begin {array}{cc}\alpha & \beta \\ \gamma & x\end {array}\right)$ have the same value $F_{1}=\frac {2\alpha }{2\alpha +\beta +\gamma }$ regardless of the value of *x*, while the corresponding MCC values range from $-\sqrt {\frac {\beta \gamma }{(\alpha +\beta)(\alpha +\gamma)}}$ for *x*=0 to the asymptotic $\frac {a}{\sqrt {(\alpha +\beta)(\alpha +\gamma)}}$ for *x*→*∞*. For example, if we consider only the 63 001 confusion matrices of datasets of size 500 where TP=TN, the Pearson correlation coefficient between *F*_1_ and MCC increases to 0.9542254.

Overall, accuracy, *F*_1_, and MCC show reliable concordant scores for predictions that correctly classify both positives and negatives (having therefore many TP and TN), and for predictions that incorrectly classify both positives and negatives (having therefore few TP and TN); however, these measures show discordant behaviors when the prediction performs well just with one of the two binary classes. In fact, when a prediction displays many true positives but few true negatives (or many true negatives but few true positives) we will show that *F*_1_ and accuracy can provide misleading information, while MCC always generates results that reflect the overall prediction issues.

## Results and discussion

### Use cases

After having introduced the mathematical foundations of MCC, accuracy, and F_1_ score, and having explored their relationships, here we describe some synthetic, realistic scenarios where MCC results are more informative and truthful than the other two measures analyzed.

**Fig. 2:**
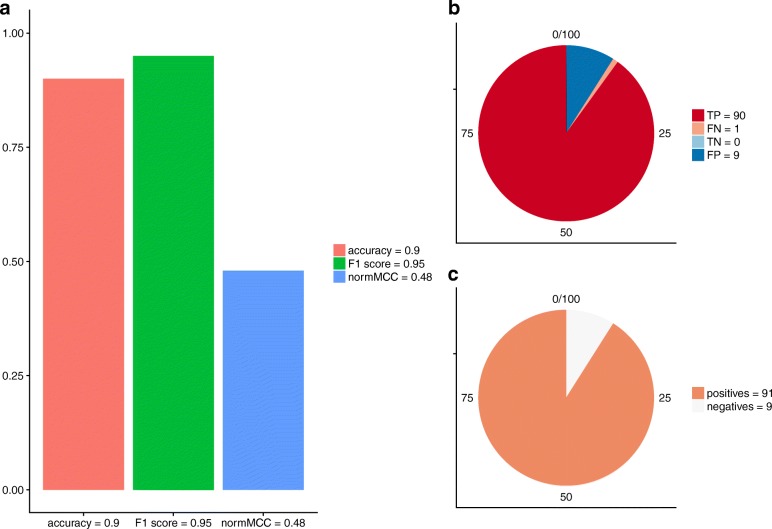
Use case A1 — Positively imbalanced dataset. **a** Barplot representing accuracy, F_1_, and normalized Matthews correlation coefficient (*normMCC* = (*MCC* + 1) / 2), all in the [0, 1] interval, where 0 is the worst possible score and 1 is the best possible score, applied to the Use case A1 positively imbalanced dataset. **b** Pie chart representing the amounts of true positives (TP), false negatives (FN), true negatives (TN), and false positives (FP). **c** Pie chart representing the dataset balance, as the amounts of positive data instances and negative data instances

**Positively imbalanced dataset — Use case A1**. Consider, for a clinical example, a positively imbalanced dataset made of 9 healthy individuals (negatives =9*%*) and 91 sick patients (positives =91*%*) (Fig. 2c). Suppose the machine learning classifier generated the following confusion matrix: TP=90, FN=1, TN=0, FP=9 (Fig. 2b).

In this case, the algorithm showed its ability to predict the positive data instances (90 sick patients out of 91 were correctly predicted), but it also displayed its lack of talent in identifying healthy controls (only 1 healthy individual out of 9 was correctly recognized) (Fig. 2b). Therefore, the overall performance should be judged poor. However, accuracy and of F_1_ showed high values in this case: *accuracy* = 0.90 and *F*_1_ *score* = 0.95, both close to the best possible value 1.00 in the [0, 1] interval (Fig. 2a). At this point, if one decided to evaluate the performance of this classifier by considering only accuracy and F_1_ score, he/she would overoptimistically think that the computational method generated excellent predictions.

Instead, if one decided to take advantage of the Matthews correlation coefficient in the Use case A1, he/she would notice the resulting MCC = –0.03 (Fig. 2a). By seeing a value close to zero in the [–1, +1] interval, he/she would be able to understand that the machine learning method has performed poorly.

**Positively imbalanced dataset — Use case A2**. Suppose the prediction generated this other confusion matrix: TP = 5, FN = 70, TN = 19, FP = 6 (Additional file 1b).

Here the classifier was able to correctly predict negatives (19 healthy individuals out of 25), but was unable to correctly identify positives (only 5 sick patients out of 70). In this case, all three statistical rates showed a low score which emphasized the deficiency in the prediction process (*accuracy* = 0.24, *F*_1_ *score* = 0.12, and *MCC* = −0.24).

**Balanced dataset — Use case B1**. Consider now, as another example, a balanced dataset made of 50 healthy controls (negatives =50*%*) and 50 sick patients (positives =50*%*) (Additional file 2c). Imagine that the machine learning prediction generated the following confusion matrix: TP=47, FN=3, TN=5, FP=45 (Additional file 2b).

Once again, the algorithm exhibited its ability to predict the positive data instances (47 sick patients out of 50 were correctly predicted), but it also demonstrated its lack of talent in identifying healthy individuals (only 5 healthy controls of 50 were correctly recognized) (Additional file 2b). Again, the overall performance should be considered mediocre.

Checking only F_1_, one would read a good value (0.66 in the [0, 1] interval), and would be overall satisfied about the prediction (Additional file 2a). Once again, this score would hide the truth: the classification algorithm has performed poorly on the negative subset. The Matthews correlation coefficient, instead, by showing a score close to random guessing (+0.07 in the [–1, +1] interval) would be able to inform that the machine learning method has been on the wrong track. Also, it is worth noticing that accuracy would provide with an informative result in this case (0.52 in the [0, 1] interval).

**Balanced dataset — Use case B2**. As another example, imagine the classifier produced the following confusion matrix: TP = 10, FN = 40, TN = 46, FP = 4 (Additional file 3b).

Similar to what happened for the Use case A2, the method was able to correctly predict many negative cases (46 healthy individuals out of 50), but failed in predicting most of positive data instances (only 10 sick patients were correctly predicted out of 50). Like for the Use case A2, accuracy, F_1_ and MCC show average or low result scores (*accuracy* = 0.56, *F*_1_ *score* = 0.31, and *MCC* = +0.17), correctly informing you about the non-optimal performance of the prediction method (Additional file 3a).

**Negatively imbalanced dataset — Use case C1**. As another example, analyze now this imbalanced dataset made of 90 healthy controls (negatives =90*%*) and 10 sick patients (positives =10*%*) (Additional file 4c).

Assume the classifier prediction produced this confusion matrix: TP = 9, FN = 1, TN = 1, FP = 89 (Additional file 4b).

In this case, the method revealed its ability to predict positive data instances (9 sick patients out of 10 were correctly predicted), but it also has shown its lack of skill in identifying negative cases (only 1 healthy individual out of 90 was correctly recognized) (Additional file 4c). Again, the overall performance should be judged modest.

Similar to the Use case A2 and *B2*, all three statistical scores generated low results that reflect the mediocre quality of the prediction: *F*_1_ *score* = 0.17 and *accuracy* = 0.10 in the [0, 1] interval, and *MCC* = −0.19 in the [–1, +1] interval (Additional file 4a).

**Negatively imbalanced dataset — Use case C2**. As a last example, suppose you obtained this alternative confusion matrix, through another prediction: TP = 2, FN = 9, TN = 88, FP = 1 (Additional file 5b).

Similar to the Use case A1 and B1, the method was able to correctly identify multiple negative data instances (88 healthy patients out of 89), but unable to correctly predict most of sick patients (only 2 true positives out of 11 possible elements).

Here, accuracy showed a high value: 0.90 in the [0, 1] interval.

On the contrary, if one decided to take a look at F_1_ and at the Matthews correlation coefficient, by noticing low values value (*F*_1_ *score* = 0.29 in the [0, 1] interval and *MCC* = +0.31 in the [–1, +1] interval), she/he would be correctly informed about the low quality of the prediction (Additional file 5a).

As we explained earlier, the key advantage of the Matthews correlation coefficient is that it generates a high quality score *only* if the prediction correctly classified a high percentage of negative data instances *and* a high percentage of positive data instances, with any class balance or imbalance.

**Recap**. We recap here the results obtained for the six use cases (Table 4). For the Use case A1 (negatively imbalanced dataset), the machine learning classifier was unable to correctly predict negative data instances, and it therefore produced confusion matrices featuring few true negatives (TN). There, accuracy and F_1_ generated overoptimistic and inflated results, while the Matthews correlation coefficient was the only statistical rate which identified the aforementioned prediction problem, and therefore to provide a low truthful quality score.

**Table 4:** Recap of the six use cases results

	Balance		Confusion matrix				Accuracy [0, 1]	F_1_ score [0, 1]	MCC [–1, +1]	Figure	Informative
	Pos	Neg	TP	FN	TN	FP					Response
Use case A1 Positively imbalanced dataset	91	9	90	1	0	9	0.90	0.95	**–0.03**	Figure 2	**MCC**
Use case A2 Positively imbalanced dataset	75	25	5	70	19	6	**0.24**	**0.12**	**–0.24**	Suppl. Additional file 1	**Accuracy, F**_**1**_** score, MCC**
Use case B1 Balanced dataset	50	50	47	3	5	45	**0.52**	0.66	**+0.07**	Suppl. Additional file 2	**Accuracy, MCC**
Use case B2 Balanced dataset	50	50	10	40	46	4	**0.56**	**0.31**	**+0.17**	Suppl. Additional file 3	**accuracy, F**_**1**_** score, MCC**
Use case C1 Negatively imbalanced dataset	10	90	9	1	1	89	**0.10**	**0.17**	**–0.19**	Suppl. Additional file 4	**accuracy, F**_**1**_** score, MCC**
Use case C2 Negatively imbalanced dataset	11	89	2	9	88	1	0.90	**0.29**	**+0.31**	Suppl. Additional file 5	**F**_**1**_** score, MCC**

For the Use case A1, MCC is the only statistical rate able to truthfully inform the readership about the poor performance of the classifier. For the Use case B1, MCC and accuracy are able to inform about the poor performance of the classifier in the prediction of negative data instances, while for the Use case A2, B2, C1, all the three rates (accuracy, F_1_, and MCC) are able to show this information. For the Use case C2, the MCC and F_1_ are able to recognize the weak performance of the algorithm in predicting one of the two original dataset classes. pos: number of positives. neg: number of negatives. TP: true positives. FN: false negatives. TN: true negatives. FP: false positives. Informative response: list of confusion matrix rates able to reflect the poor performance of the classifier in the prediction task. We highlighted in bold the informative response of each use case

In the Use case A2 (positively imbalanced dataset), instead, the method did not predict correctly enough positive data instances, and therefore showed few true positives. Even if accuracy showed an excessively high result score, the values of F_1_ and MCC correctly reflected the low quality of the prediction.

In the Use case B1 (balanced dataset), the machine learning method was unable to correctly predict negative data instances, and therefore produced a confusion matrix featuring few true negatives (TN). In this case, F_1_ generated an overoptimistic result, while accuracy and the MCC correctly produced low results that highlight an issue in the prediction.

The classifier did not find enough true positives for the Use case B2 (balanced dataset), too. In this case, all the analyzed rates (accuracy, F_1_, and MCC) produced average or low results which correctly represented the prediction issue.

Also in the Use case C1 (positively imbalanced dataset), the machine learning method was unable to correctly recognize negative data instances, and therefore produced a confusion matrix with a low number of true negative (TN). Here, accuracy again generated an overoptimistic inflated score, while F_1_ and the MCC correctly produced low results that indicated a problem in the prediction process.

Finally, in the last Use case C2 (positively imbalanced dataset), the prediction technique failed in predicting negative elements, and therefore its confusion matrix showed a low percentage of true negatives. Here accuracy again generated overoptimistic, misleading, and inflated high results, while F_1_ and MCC were able to produce a low score that correctly reflected the prediction issue.

In summary, even if F_1_ and accuracy results were able to reflect the prediction issue in some of the six analyzed use cases, the Matthews correlation coefficient was the only score which correctly indicated the prediction problem in all six examples (Table 4).

Particularly, in the Use case A1 (a prediction which generated many true positives and few true negatives on a positively imbalanced dataset), the MCC was the only statistical rate able to truthfully highlight the classification problem, while the other two rates showed misleading results (Fig. 2).

These results show that, while accuracy and F_1_ score often generate high scores that do not inform the user about ongoing prediction issues, the MCC is a robust, useful, reliable, truthful statistical measure able to correctly reflect the deficiency of any prediction in any dataset.

### Genomics scenario: colon cancer gene expression

In this section, we show a real genomics scenario where the Matthews correlation coefficient result being more informative than accuracy and F_1_ score.

**Dataset**. We trained and applied several machine learning classifiers to gene expression data from the microarray experiments of colon tissue released by Alon et al. [103] and made it publically available within the Partial Least Squares Analyses for Genomics (*plsgenomics*) R package [104, 105]. The dataset contains 2,000 gene probsets for 62 patients, of which 22 are healthy controls and 40 have colon cancer (35.48% negatives and 64.52% positives) [106].

**Experiment design**. We employed machine learning binary classifiers to predict patients and healthy controls in this dataset: gradient boosting [107], decision tree [108], *k*-nearest neighbors (*k*-NN) [109], support vector machine (SVM) with linear kernel [7], and support vector machine with radial Gaussian kernel [7].

For gradient boosting and decision tree, we trained the classifiers on a training set containing 80% of randomly selected data instances, and test them on the test set containing the remaining 20% data instances. For *k*-NN and SVMs, we split the dataset into training set (60% data instances, randomly selected), validation set (20% data instances, randomly selected), and the test set (remaining 20% data instances). We used the validation set for the hyper-parameter optimization grid search [97]: number *k* of neighbors for *k*-NN, and cost *C* hyper-parameter for the SVMs. We trained each model having a different hyper-parameter on the training set, applied it to the validation set, and then picked the one obtaining the highest MCC as final model to be applied to the test set. For all the classifiers, we repeated the experiment execution ten times and recorded the average results for MCC, F_1_ score, accuracy, true positive (TP) rate, and true negative (TN) rate.

We then ranked the results obtained on the test sets or the validation sets first based on the MCC, then based on the F_1_ score, and finally based on the accuracy (Table 5).

**Table 5:** Colon cancer prediction rankings

Classifier	MCC	F_1_ score	Accuracy	TP rate	TN rate
MCC ranking:					
Gradient boosting	**+0.55**	0.81	0.78	0.85	0.69
Decision tree	**+0.53**	0.82	0.77	0.88	0.58
*k*-nearest neighbors	**+0.48**	0.87	0.80	0.92	0.52
Linear SVM	**+0.41**	0.82	0.76	0.86	0.53
Radial SVM	**+0.29**	0.75	0.67	0.86	0.40
F_1_ score ranking:					
*k*-nearest neighbors	+0.48	**0.87**	0.80	0.92	0.52
Linear SVM	+0.41	**0.82**	0.76	0.86	0.53
Decision tree	+0.53	**0.82**	0.77	0.88	0.58
Gradient boosting	+0.55	**0.81**	0.78	0.85	0.69
Radial SVM	+0.29	**0.75**	0.67	0.86	0.40
Accuracy ranking:					
*k*-nearest neighbors	+0.48	0.87	**0.80**	0.92	0.52
Gradient boosting	+0.55	0.81	**0.78**	0.85	0.69
Decision tree	+0.53	0.82	**0.77**	0.88	0.58
Linear SVM	+0.41	0.82	**0.76**	0.86	0.53
Radial SVM	+0.29	0.75	**0.67**	0.86	0.40

Prediction results on colon cancer gene expression dataset, based on MCC, F_1_ score, and accuracy. linear SVM: support vector machines with linear kernel. MCC: worst value –1 and best value +1. F_1_ score, accuracy, TP rate, and TN rate: worst value 0 and best value 1. To avoid additional complexity and keep this table simple to read, we prefered to exclude the standard deviation of each result metric. We highlighted in bold the ranking of each rate

**Results: different metric, different ranking**. The three rankings we employed to report the same results (Table 5) show two interesting aspects. First, the top classifier changes when we consider the ranking based on MCC, F_1_ score, or accuracy. In the MCC ranking, in fact, the top performing method is gradient boosting (MCC = +0.55), while in the F_1_ score ranking and in the accuracy ranking the best classifier resulted being *k*-NN (F_1_ score = 0.87 and accuracy = 0.81). The ranks of the other methods change, too: linear SVM is ranked forth in the MCC ranking and in the accuracy ranking, but ranked second in the F_1_ score ranking. Decision tree changes its position from one ranking to another, too.

As mentioned earlier, for binary classifications like this, we prefer to focus on the ranking obtained by the MCC, because this rate generates a high score only if the classifier was able to correctly predict the majority of the positive data instances and the majority of the negative data instances. In our example, in fact, the top MCC ranking classifier gradient boosting did quite well both on the recall (TP rate = 0.85) and on the specificity (TN rate = 0.69). *k*-NN, that is the top performing method both in the F_1_ score ranking and in the accuracy ranking, instead, obtained an excellent score for recall (TP rate = 0.92) but just sufficient on the specificity (TN rate = 0.52).

The F_1_ score ranking and the accuracy ranking, in conclusion, are hiding this important flaw of the top classifier: *k*-NN was unable top correctly predict a high percentage of patients. The MCC ranking, instead, takes into account this information.

**Results: F**_**1**_** score and accuracy can mislead, but MCC does not**. The second interesting aspect of the results we obtained relates to the radial SVM (Table 5). If a researcher decided to evaluate the performance of this method by observing only the F_1_ score and the accuracy, she/he would notice good results (F_1_ score = 0.75 and accuracy = 0.67) and might be satisfied about them. These results, in fact, mean 3/4 correct F_1_ score and 2/3 correct accuracy.

However, these values of F_1_ score and accuracy would mislead the researcher once again: with a closer look to the results, one can notice that the radial SVM has performed poorly on the true negatives (TN rate = 0.40), by correctly predicting less than half patients. Similar to the synthetic Use case A1 previously described (Fig. 2 and Table 4), the Matthews correlation coefficient is the only aggregate rate highlighting the weak performance of the classifier here. With its low value (MCC = +0.29), the MCC informs the readers about the poor general outcome of the radial SVM, while the accuracy and F_1_ score show misleading values.

## Conclusions

Scientists use confusion matrices to evaluate binary classification problems; therefore, the availability of a unified statistical rate that is able to correctly represent the quality of a binary prediction is essential. Accuracy and F_1_ score, although popular, can generate misleading results on imbalanced datasets, because they fail to consider the ratio between positive and negative elements. In this manuscript, we explained the reasons why Matthews correlation coefficient (MCC) can solve this issue, through its mathematical properties that incorporate the dataset imbalance and its invariantness for class swapping. The criterion of MCC is intuitive and straightforward: to get a high quality score, the classifier has to make correct predictions both on the majority of the negative cases, and on the majority of the positive cases, independently of their ratios in the overall dataset. F_1_ and accuracy, instead, generate reliable results only when applied to balanced datasets, and produce misleading results when applied to imbalanced cases. For these reasons, we suggest all the researchers working with confusion matrices to evaluate their binary classification predictions through the MCC, instead of using F_1_ score or accuracy.

Regarding the limitations of this comparative article, we recognize that additional comparisons with other rates (such as Cohen’s Kappa [70], Cramér’s V [37], and K measure [81]) would have provided further information about the role of MCC in binary classification evaluation. We prefered to focus on accuracy and F_1_ score, instead, because accuracy and F_1_ score are more commonly used in machine learning studies related to biomedical applications.

In the future, we plan to investigate further the relationship between MCC and Cohen’s Kappa, Cramér’s V, K measure, balanced accuracy, F macro average, and F micro average.

## Supplementary information

**Additional file 1** Use case A2 — Positively imbalanced dataset. **(a)** Barplot representing *accuracy*, F_1_* score*, and normalized *Matthews correlation coefficient* (*normMCC* = (*MCC* + 1) / 2), all in the [0, 1] interval, where 0 is the worst possible score and 1 is the best possible score, applied to the Use case A2 positively imbalanced dataset. **(b)** Pie chart representing the amounts of true positives (TP), false negatives (FN), true negatives (TN), and false positives (FP). **(c)** Pie chart representing the dataset balance, as the amounts of positive data instances and negative data instances.

**Additional file 2** Use case B1 — Balanced dataset. **(a)** Barplot representing *accuracy*, F_1_* score*, and normalized *Matthews correlation coefficient* (*normMCC* = (*MCC* + 1) / 2), all in the [0, 1] interval, where 0 is the worst possible score and 1 is the best possible score, applied to the Use case B1 balanced dataset. **(b)** Pie chart representing the amounts of true positives (TP), false negatives (FN), true negatives (TN), and false positives (FP). **(c)** Pie chart representing the dataset balance, as the amounts of positive data instances and negative data instances.

**Additional file 3** Use case B2 — Balanced dataset. **(a)** Barplot representing *accuracy*, F_1_* score*, and normalized *Matthews correlation coefficient* (*normMCC* = (*MCC* + 1) / 2), all in the [0, 1] interval, where 0 is the worst possible score and 1 is the best possible score, applied to the Use case B2 balanced dataset. **(b)** Pie chart representing the amounts of true positives (TP), false negatives (FN), true negatives (TN), and false positives (FP). **(c)** Pie chart representing the dataset balance, as the amounts of positive data instances and negative data instances.

**Additional file 4** Use case C1 — Negatively imbalanced dataset. **(a)** Barplot representing *accuracy*, F_1_* score*, and normalized *Matthews correlation coefficient* (*normMCC* = (*MCC* + 1) / 2), all in the [0, 1] interval, where 0 is the worst possible score and 1 is the best possible score, applied to the Use case C1 negatively imbalanced dataset. **(b)** Pie chart representing the amounts of true positives (TP), false negatives (FN), true negatives (TN), and false positives (FP). **(c)** Pie chart representing the dataset balance, as the amounts of positive data instances and negative data instances.

**Additional file 5** Use case C2 — Negatively imbalanced dataset. **(a)** Barplot representing *accuracy*, F_1_* score*, and normalized *Matthews correlation coefficient* (*normMCC* = (*MCC* + 1) / 2), all in the [0, 1] interval, where 0 is the worst possible score and 1 is the best possible score, applied to the Use case C2 negatively imbalanced dataset. **(b)** Pie chart representing the amounts of true positives (TP), false negatives (FN), true negatives (TN), and false positives (FP). **(c)** Pie chart representing the dataset balance, as the amounts of positive data instances and negative data instances.

## Data Availability

The data and the R software code used in this study for the tests and the plots are publically available at the following web URL: https://github.com/davidechicco/MCC.

## Abbreviations

*k*-NN: *k*-nearest neighbors
AUC: Area under the curve
FDA: Food and drug administration
FN: False negatives
FP: false positives. MAQC/SEQC: MicroArray II / sequencing quality control
MCC: Matthews correlation coefficient
PCC: Pearson correlation coefficient
PLS: Partial least squares
PR: Precision-recall
ROC: Receiver operating characteristic
SVM: Support vector machine
TN: True negatives
TP: True positives
