# Supplementary figures and images for: The advantages of the Matthews correlation coefficient (MCC) over F1 score and accuracy in binary classification evaluation
Source: BMC Genomics. 2020 Jan 2;21:6. doi: 10.1186/s12864-019-6413-7 (PMC6941312; doi:10.1186/s12864-019-6413-7)

**a**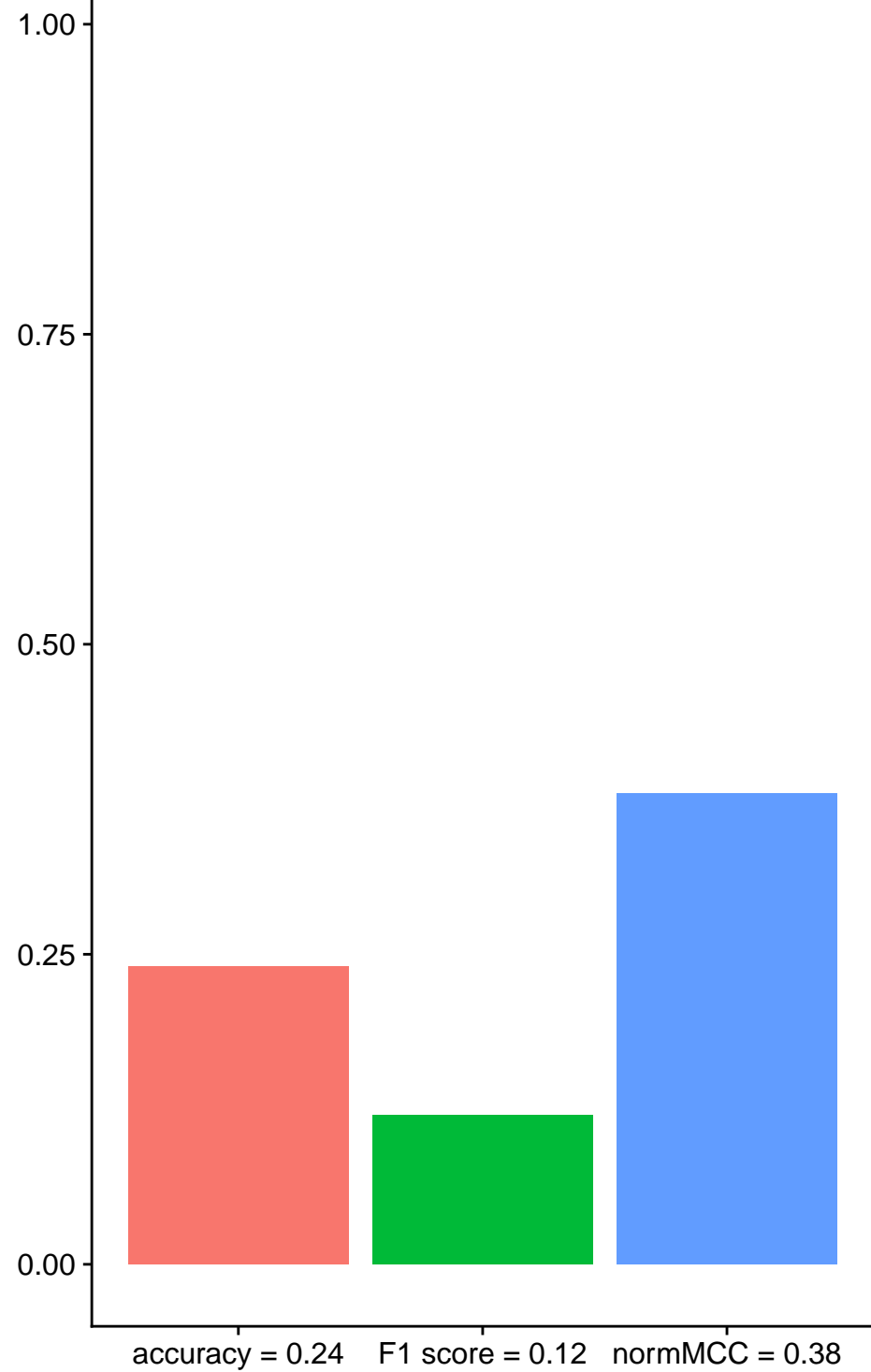**b**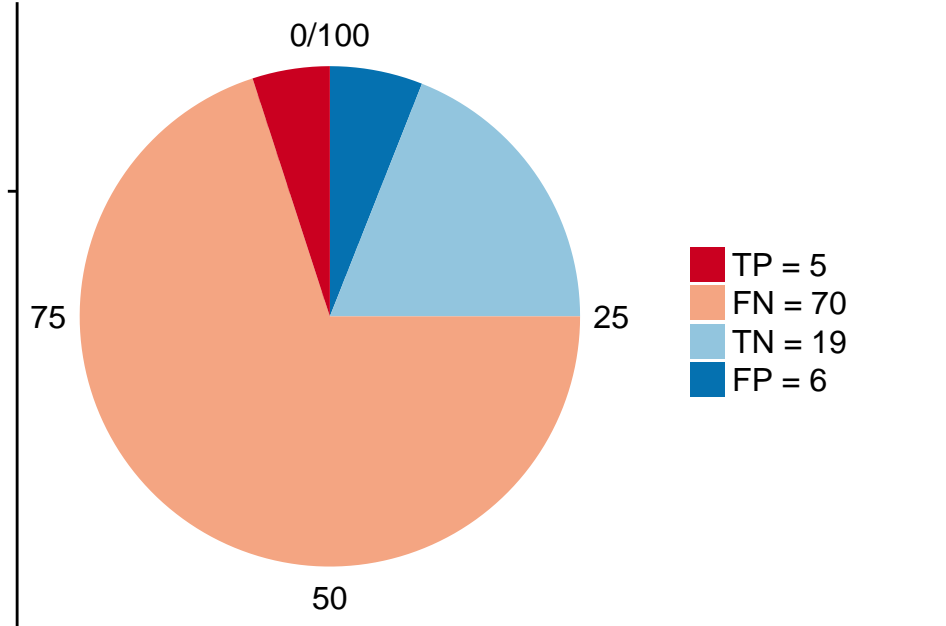**c**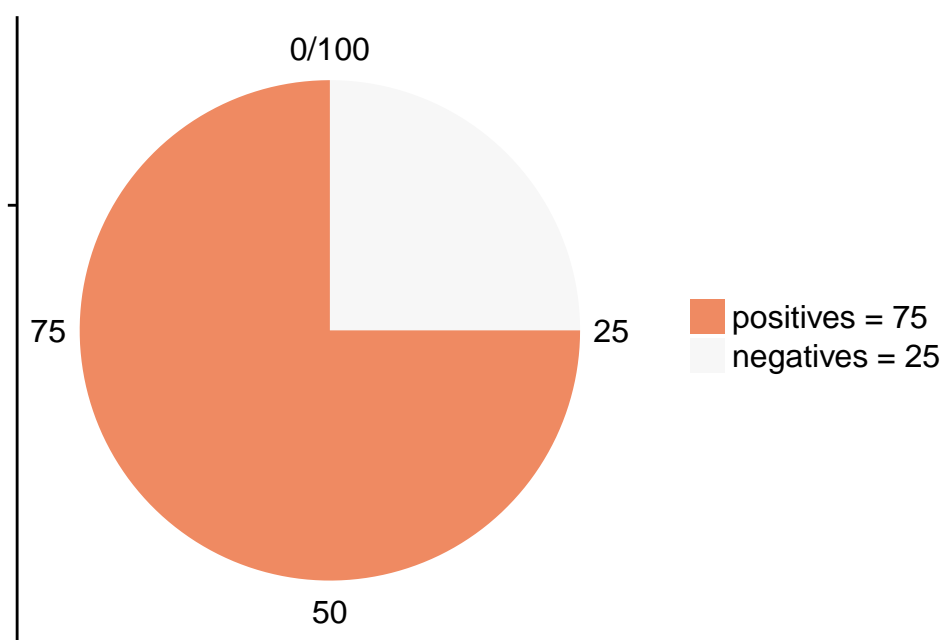

Supplement: Supplementary file 1 — Additional file 1 Use case A2 — Positively imbalanced dataset. (a) Barplot representing accuracy, F1 score, and normalized Matthews correlation coefficient (normMCC = (MCC + 1) / 2), all in the [0, 1] interval, where 0 is the worst possible score and 1 is the best possible score, applied to the Use case A2 positively imbalanced dataset. (b) Pie chart representing the amounts of true positives (TP), false negatives (FN), true negatives (TN), and false positives (FP). (c) Pie chart representing the dataset balance, as the amounts of positive data instances and negative data instances. [file 12864_2019_6413_MOESM1_ESM.pdf]

**a**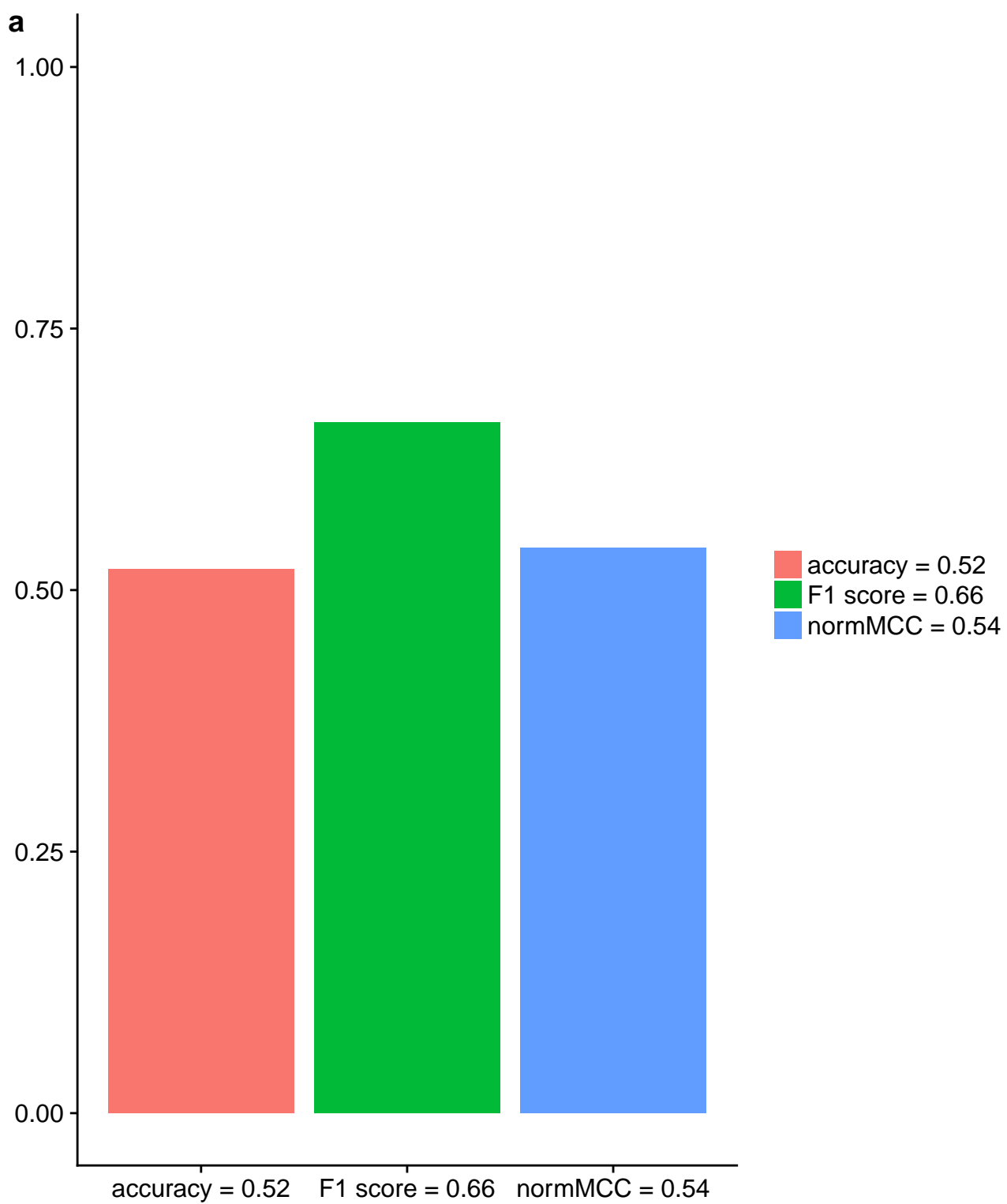**b**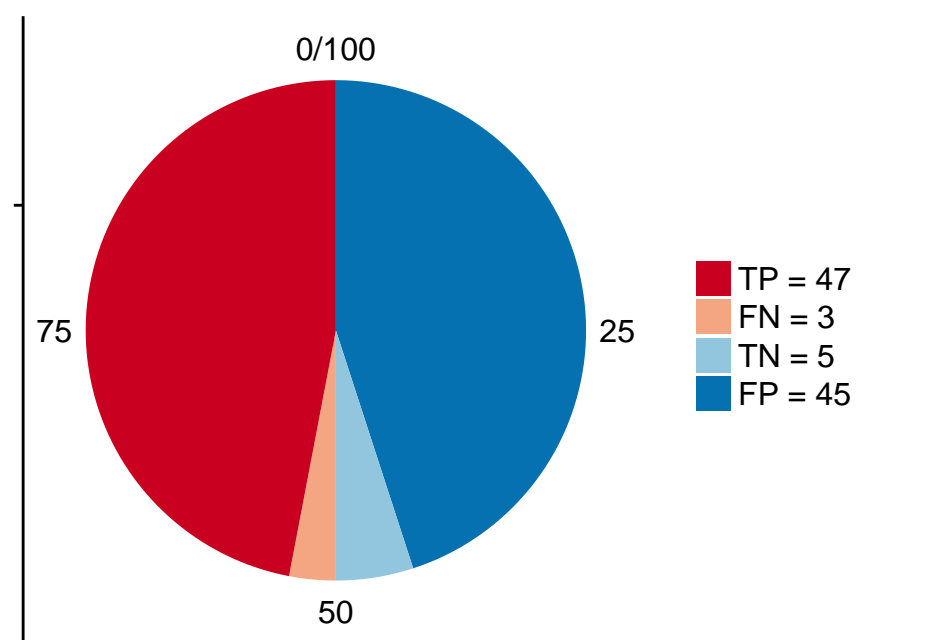**c**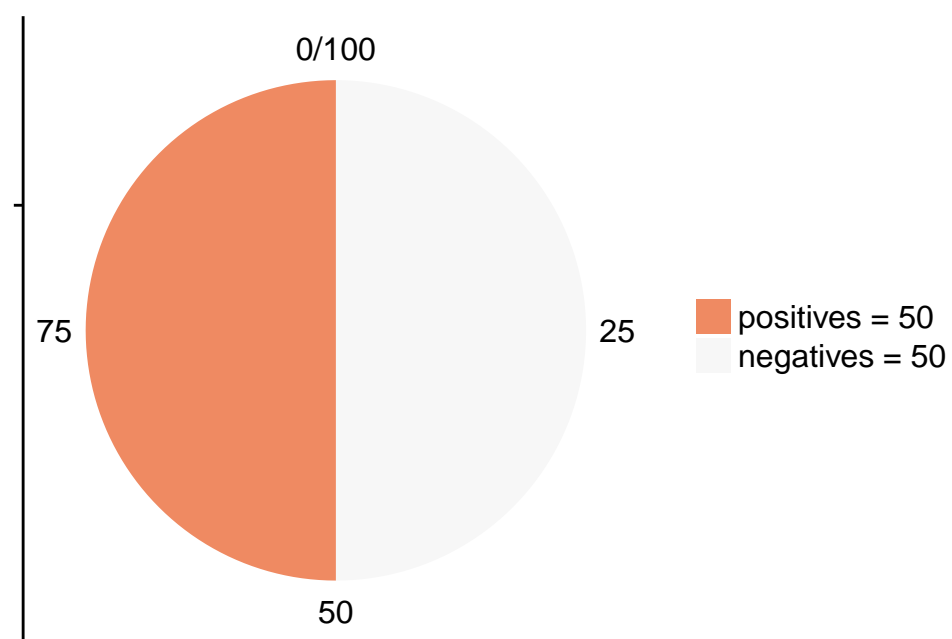

Supplement: Supplementary file 2 — Additional file 2 Use case B1 — Balanced dataset. (a) Barplot representing accuracy, F1 score, and normalized Matthews correlation coefficient (normMCC = (MCC + 1) / 2), all in the [0, 1] interval, where 0 is the worst possible score and 1 is the best possible score, applied to the Use case B1 balanced dataset. (b) Pie chart representing the amounts of true positives (TP), false negatives (FN), true negatives (TN), and false positives (FP). (c) Pie chart representing the dataset balance, as the amounts of positive data instances and negative data instances. [file 12864_2019_6413_MOESM2_ESM.pdf]

**a**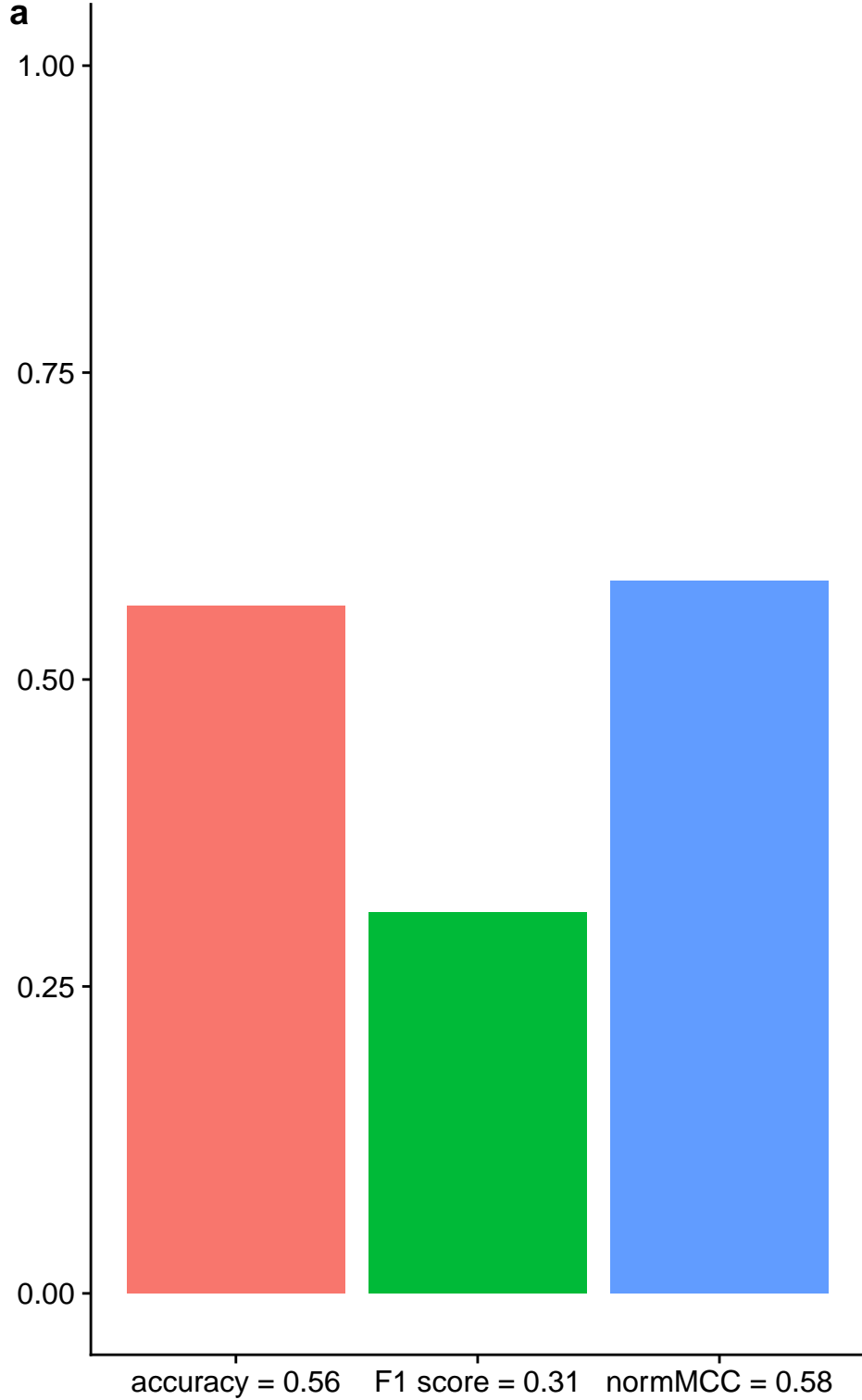**b**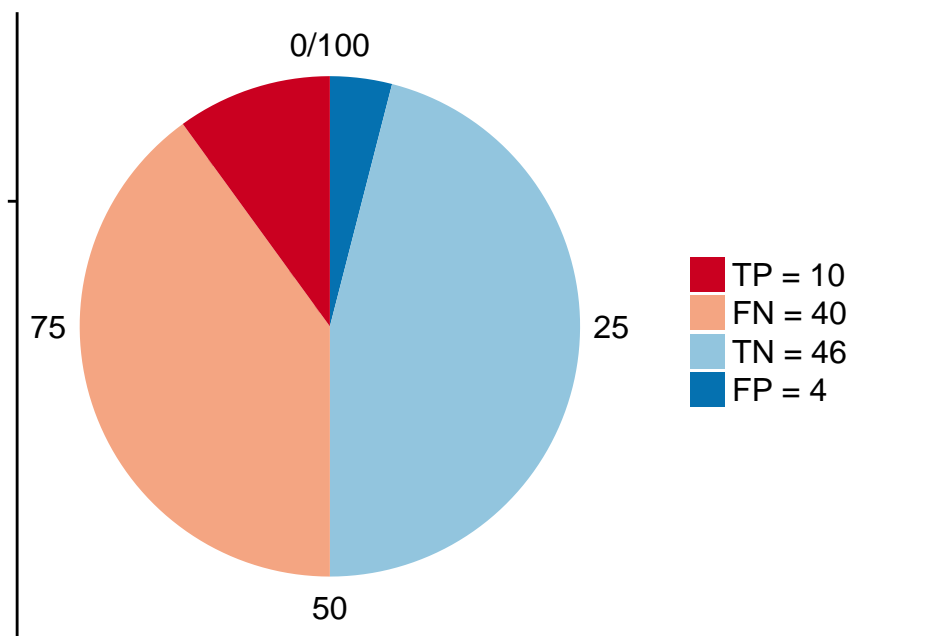**c**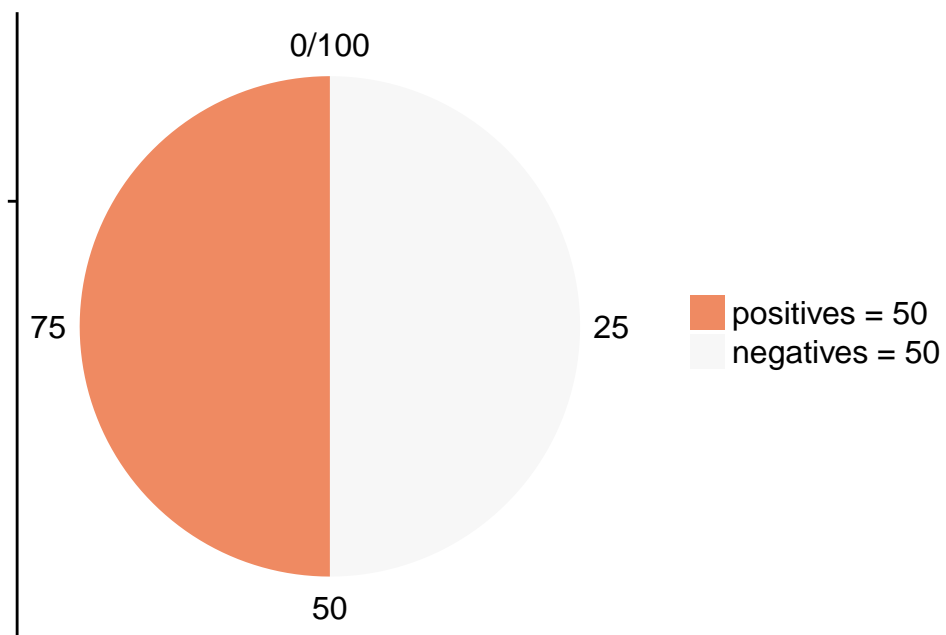

Supplement: Supplementary file 3 — Additional file 3 Use case B2 — Balanced dataset. (a) Barplot representing accuracy, F1 score, and normalized Matthews correlation coefficient (normMCC = (MCC + 1) / 2), all in the [0, 1] interval, where 0 is the worst possible score and 1 is the best possible score, applied to the Use case B2 balanced dataset. (b) Pie chart representing the amounts of true positives (TP), false negatives (FN), true negatives (TN), and false positives (FP). (c) Pie chart representing the dataset balance, as the amounts of positive data instances and negative data instances. [file 12864_2019_6413_MOESM3_ESM.pdf]

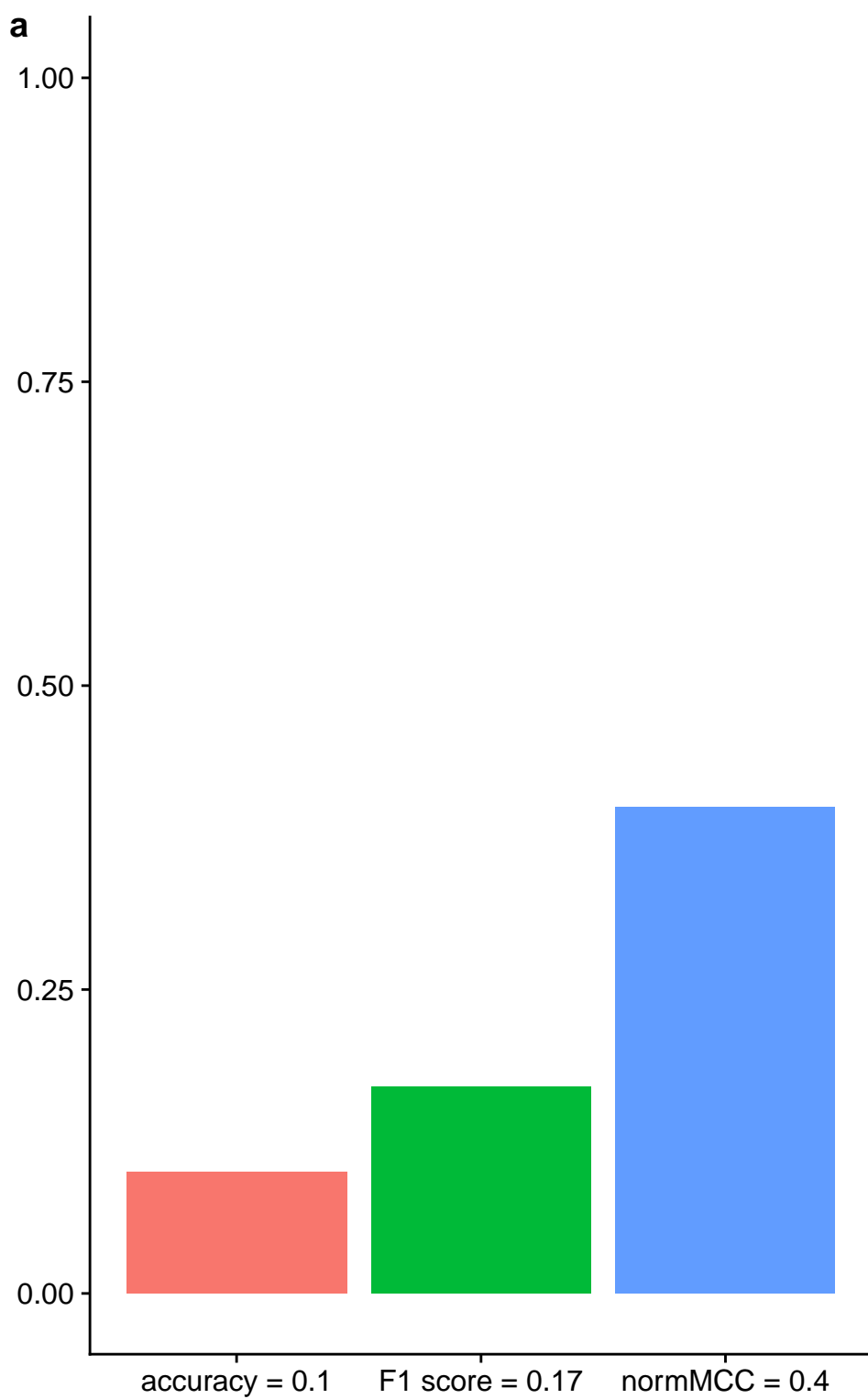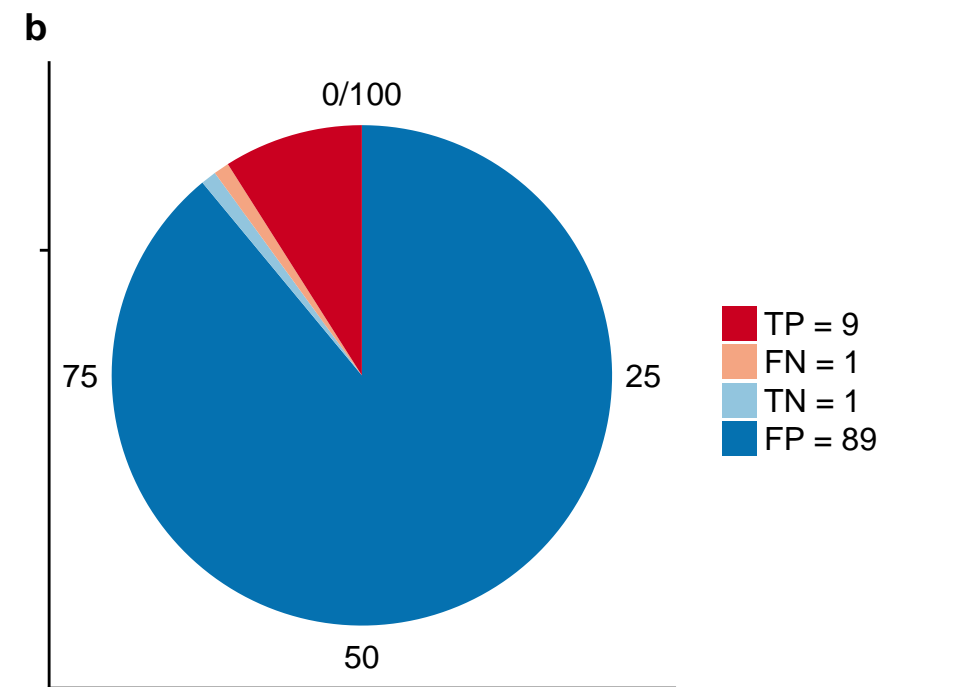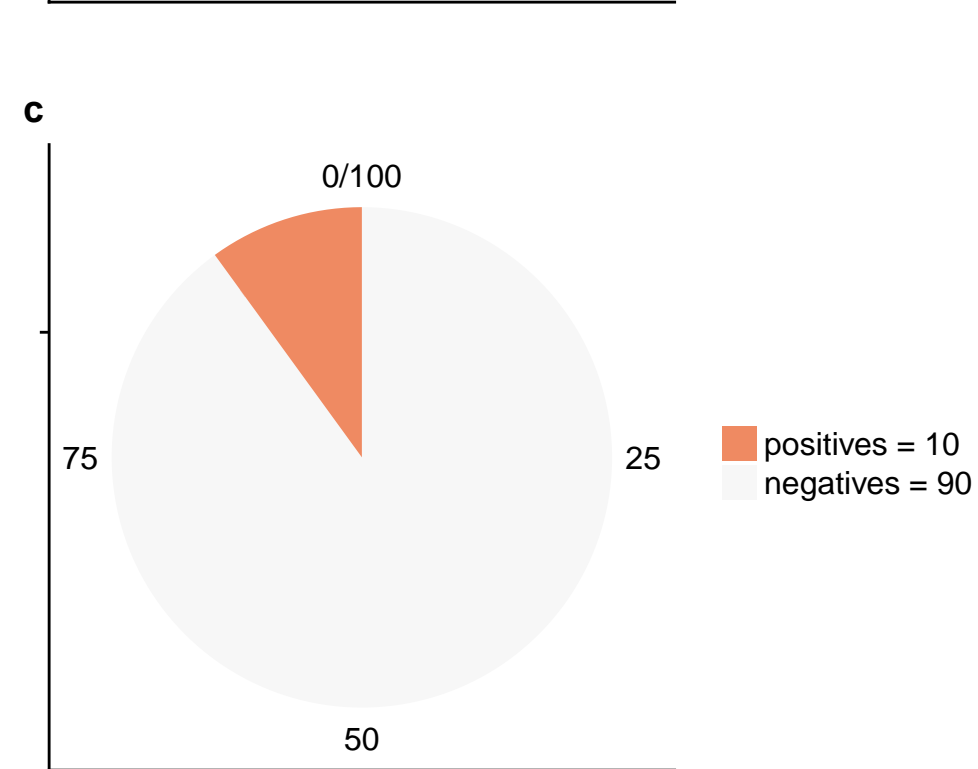

Supplement: Supplementary file 4 — Additional file 4 Use case C1 — Negatively imbalanced dataset. (a) Barplot representing accuracy, F1 score, and normalized Matthews correlation coefficient (normMCC = (MCC + 1) / 2), all in the [0, 1] interval, where 0 is the worst possible score and 1 is the best possible score, applied to the Use case C1 negatively imbalanced dataset. (b) Pie chart representing the amounts of true positives (TP), false negatives (FN), true negatives (TN), and false positives (FP). (c) Pie chart representing the dataset balance, as the amounts of positive data instances and negative data instances. [file 12864_2019_6413_MOESM4_ESM.pdf]

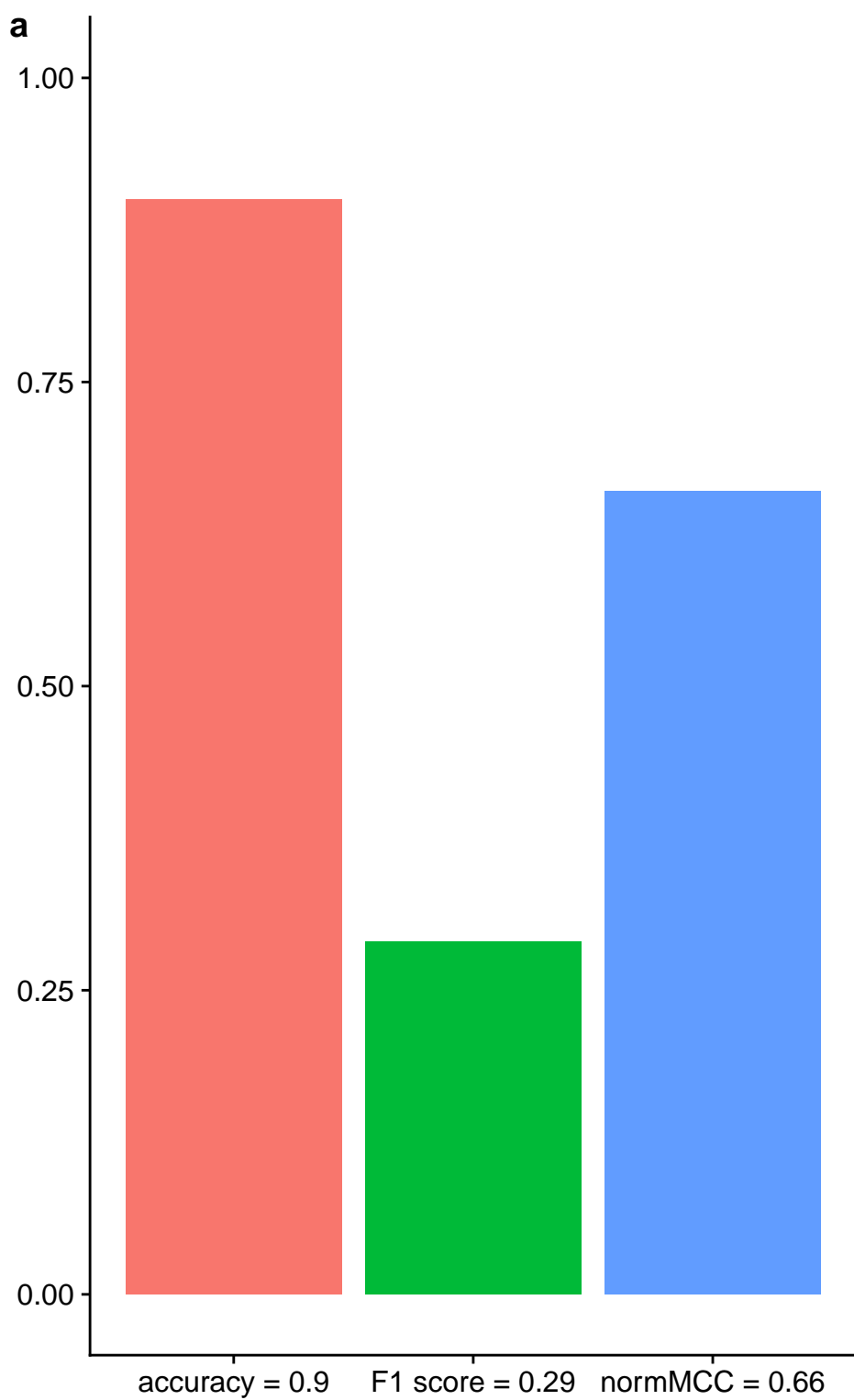

accuracy = 0.9  
F1 score = 0.29  
normMCC = 0.66

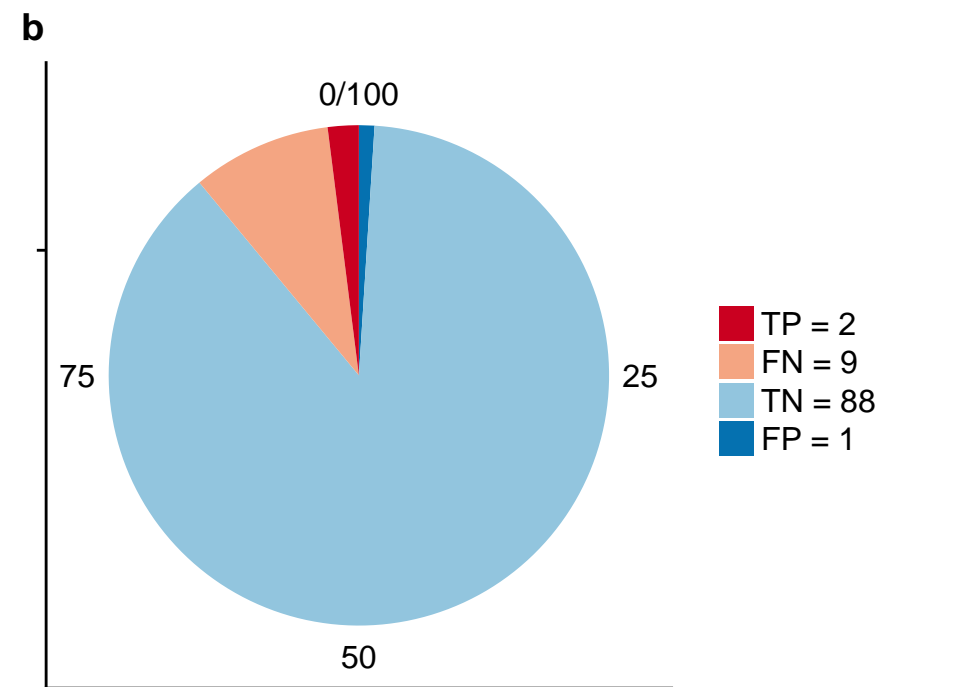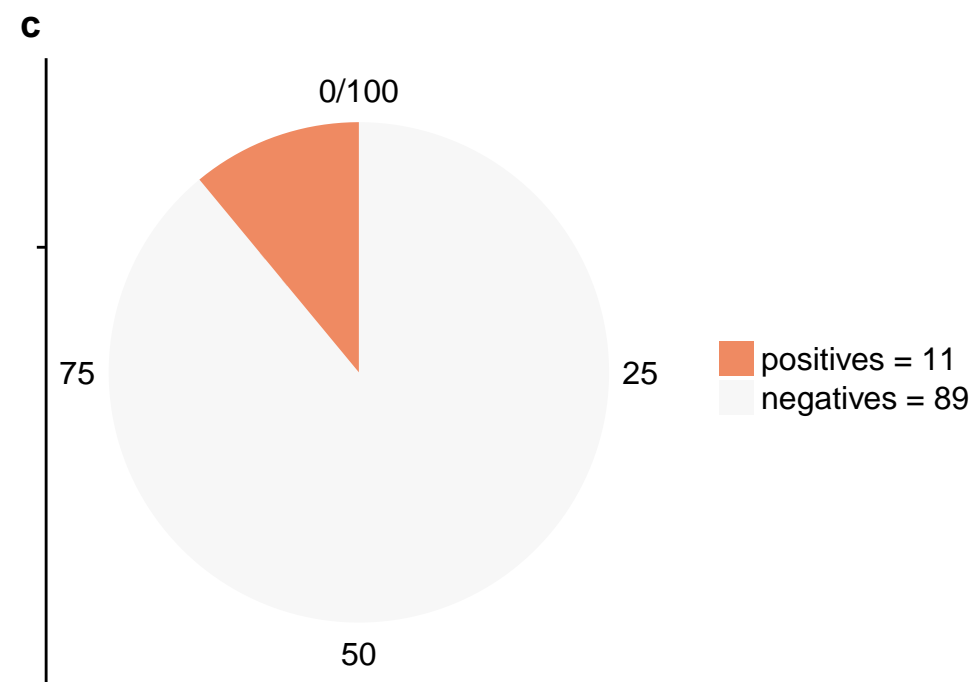

Supplement: Supplementary file 5 — Additional file 5 Use case C2 — Negatively imbalanced dataset. (a) Barplot representing accuracy, F1 score, and normalized Matthews correlation coefficient (normMCC = (MCC + 1) / 2), all in the [0, 1] interval, where 0 is the worst possible score and 1 is the best possible score, applied to the Use case C2 negatively imbalanced dataset. (b) Pie chart representing the amounts of true positives (TP), false negatives (FN), true negatives (TN), and false positives (FP). (c) Pie chart representing the dataset balance, as the amounts of positive data instances and negative data instances. [file 12864_2019_6413_MOESM5_ESM.pdf]
